# The network approach: A path to decolonize mental health care

**DOI:** 10.3389/fpubh.2023.1052077

**Published:** 2023-02-13

**Authors:** Rediet Emebet Getnet Alemu, Tom L. Osborn, Christine M. Wasanga

**Affiliations:** ^1^Shamiri Institute, Nairobi, Kenya; ^2^Department of Psychology, Kenyatta University, Nairobi, Kenya

**Keywords:** mental health, Africa, decolonize, network, de-stigmatization

## Abstract

The violent colonial history of psychiatry in Africa prevents individuals from help-seeking. Because of this history, mental health care is now stigmatized, and clinical research, practice, and policy fail to capture the salient features of distress across African communities. If we are to transform mental health care for all, we must adopt decolonizing frameworks to ensure mental health research, practice, and policy are enacted in a manner that is ethical, democratic, critical, and serves the needs of local communities. Here, we present that the network approach to psychopathology as an invaluable tool in achieving this purpose. The network approach recognizes mental health disorders not as discrete entities, but rather as dynamic networks that are made of psychiatric symptoms (called *nodes*) and the relationships between these symptoms (called *edges*). This approach can pave a path to decolonizing mental health care by alleviating stigma, allowing context-based understanding of mental health and mental health problems, opening new avenues for (low-cost) mental health care and empowering local researchers to pioneer context-based knowledge production and treatment.

## 1. Introduction

The history of “formal” or Western-derived psychiatry in Africa is, to say the least, a violent and colonial one. This form of psychiatry was introduced by colonial administrators in the 19th and early 20th centuries in their respective fledgling colonies across Africa (1). It is now clear—from recent historical analyses—that these efforts were mostly informed by a commitment to finding pseudo-scientific basis of advancing the colonial enterprise instead of providing care to Africans (1, 2). Accordingly, it's no wonder that previous efforts to study the “African mind” were highly influenced by anti-African and anti-Black racism and by white superiority.

### 1.1. The history of psychiatry in Africa: An illustration from British Kenya

To paint a picture of this history of Western-derived psychiatry in Africa, let us take for instance, the context of British Kenya, where we work. Anti-African sentiments and support for the colonial agenda are evident in the work of the foremost British psychiatrist of the colonial era, Carothers (3). In his work, Carothers concluded that all Africans have a singular culture which shaped their minds and rendered them suffering from a mental health disorder that could only be cured through “acculturation”—i.e., the practice of forcefully making Africans abandon their cultures for a Western one (3). As such, many forms of mental health care that were part of the sociocultural fabric of African life, such as the use of traditional healers, were abandoned as part of these efforts of curing Africans from their culture. Carothers, and his compatriots, displayed grossly incorrect generalizations that were evidently rooted in the notion of white superiority and the need to provide an “empirical” basis for the necessity of a colonial administration (1).

Nowhere is this agenda more evident than the colonial administration's use of psychiatry as a primary tool in making sense of decolonial and liberation movements and providing the framework to crush these movements (1). In Kenya, the British colonial government employed the services of Carothers to arrive at a psychiatric explanation for the cause of the Mau Mau uprising (3)—the movement of the Land and Freedom Army, dubbed “Mau Mau” by the colonial administration, which sought to reclaim African land that was forcefully taken for white settlement (4). Unsurprisingly, Carothers concluded that this movement arose primarily due to the Kikuyu people's culture which embodied “brutal oaths and obscene rituals” and their “‘magic' modes of thinking” (3).

In addition, the primary model of psychiatric care was the colonial asylum. Individuals, after a diagnosis of a mental health problem, could be detained in such asylums and deprived of their individual, social, and political liberties. Historians have now concluded that this was a means of political control (1) and punishment (5) by the colonial administrators. The foremost mental health care facility in Kenya, the Mathari Mental Hospital, was founded as one such colonial asylum and aptly named the “Nairobi Lunatic Asylum” upon its establishment in 1910 (6). Unsurprisingly, its patients were exclusively African and its staff exclusively European (6).

### 1.2. Consequences of the history of mental health care in Africa

Two consequences of the history of psychiatry in Africa still limit efforts to transform mental health care for all in the continent. The first is the societal stigma against help-seeking, and the second is the over-reliance on Western-derived classifications and assumptions that fail to capture the salient features of mental health and mental health problems across African communities.

#### 1.2.1. Societal stigma

In many African countries, societal stigma around mental health problems prevents many people from help-seeking and inhibits efforts to transform mental health for all (7). In Kenya, we have found that youths—as young as children in primary schools—have stigmatizing attitudes toward individuals diagnosed with mental health disorders (8). This stigma, of course, prevents both youths and adults from help-seeking (8, 9).

One reason for this stigma is undoubtedly the colonial history of psychiatry across Africa. In Kenya, for instance, the Mathari Mental Hospital still operates as the principal psychiatric facility (6). Of course, the hospital's violent and colonial history can not only prevent Kenyans from seeking help there, but also encourage them to associate Mathari with problems that require detainment. Here, it is important to note that stigma is fostered and exhibited differently in different contexts, even though stigma itself can exist anywhere. Accordingly, one can hypothesize that this stigma amongst Kenyan communities is heavily exacerbated by the local history of colonial psychiatry.

#### 1.2.2. Western-derived classifications

Clinical psychology and psychopathology have utilized the perspectives of predominantly white- and male-populations as its foundation (7, 10, 11). Psychopathology is still heavily defined through Western-derived taxonomies and classification. For instance, how we conceptualize depression (or Major Depressive Disorder) remains heavily influenced by the American psychiatric school of thought (i.e., the Feighner criteria (12)) which is now considered the gold-standard in clinical practice and research (13). Consequently, mental health care relies on assumptions that Western nosology of disorders are globally generalizable. This is an assumption that years of cross-cultural mental health research has challenged (14–17).

If some of these Western-derived taxonomies appear alien or foreign to African communities, it may inhibit help-seeking. In fact, one recent study in Kenya found that when mental health problems were conceptualized and described through a Western-derived lens, then individuals would be less likely to seek help (17). Specifically, if someone was given a formal DSM-5 diagnosis of Major Depressive Disorder (MDD), they would be less likely to accept that diagnosis and seek help than if the practitioner were to conceptualize the disorder in more specific cultural terms and prioritize the specific challenges faced by the patient i.e., extreme sadness, lack of motivation, problems with sleep, etc. (17).

## 2. The move to decolonize mental health care in Africa

The traumatic history of colonialism—and apartheid in the context of South Africa—has significant contemporary effects on how individuals and communities access and interface with mental health care (2). Therefore, decolonizing mental health care is very important to address global mental health inequity. In this context, decolonization is defined through efforts to understand, reckon with and rectify the current effects of colonialism (2). These efforts can allow us to engage with African and/or localized conceptions of mental health (18, 19). Indeed, and as is argued elsewhere, decolonizing mental health care will allow us to move toward a paradigm in which mental health research, practice, and policy is enacted in a manner that is ethical, democratic, critical, and serves the needs of local communities (20).

One approach to decolonizing mental health care in Africa is the move toward a critical and “context-based” approach to mental health (2, 21, 22). This approach pairs mental health research and practice with efforts to “promote the participation and collective action of marginalized groups” and foster knowledge production of challenges faced and ideas proposed by local communities (2). In doing so, mental health research can be informed by and address past and present injustices of its local context (2). This approach, it is argued, will ensure that mental health care remains relevant and attuned to the needs of contemporary African contexts (2).

Here, we present the network approach to psychopathology as another pathway toward decolonizing mental health (23, 24).

### 2.1. The network approach as a path toward decolonizing mental health care in Africa

In the past decade, the network approach to psychopathology has emerged as an increasingly popular framework for conceptualization of mental health disorders. In this approach, we can visualize psychiatry constructs as a dynamic system made up of symptoms and the associations between pairs of symptoms (23–25). Thus, a mental disorder is not a discrete entity but rather a network of interrelated symptoms. The approach departs from the traditional disease model where it is assumed that a disorder arises from an underlying latent disorder but rather as interactions of unique and non-overlapping variables (23–25).

In other words, the network approach represents mental health disorders, not as a discrete entity like MDD, but rather as a network that is sustained by interrelationships among the specific symptoms (23, 24, 26). Thus, “instead of being effects of a common cause, psychiatric symptoms have been argued to cause each other” (23). In this conceptualization, mental health disorders are imagined as dynamic systems that are made of psychiatric symptoms (called *nodes*) and the relationships between these symptoms (called *edges*) (24).

The network approach makes it possible to identify the most central nodes in a network, effectively identifying the symptom(s) with the highest influence on the other symptoms in the network (27). Such a symptom is identified by analyzing the number of symptoms connected to it (its edges) and the likelihood of the connected symptoms (nodes) being activated as a result of its activation (27). Accordingly, in a given context, the network approach can map out not only the psychiatric symptoms that are present, but also the relationships between these symptoms and the most strongly interconnected symptom (27). This is particularly important since interventions targeting the strongest symptom (node) in a network (a representation of interconnected symptoms) are more likely to be effective in treating the network (27). In other words, interventions which can reduce the intensity of the most central node will also reduce the intensity of all the other nodes connected to it, effectively reducing the intensity of the network as a whole.

The network approach to psychopathology can help decolonize mental health care in Africa by: (1) alleviating stigma toward mental health problems, (2) allowing context-based understanding of mental health problems, (3) opening alternative avenues for mental health care, and (4) encouraging local researchers to pioneer context-based knowledge production and treatment design.

Firstly, since this approach emphasizes the role of edges (symptom-symptom relationships) in causing mental health problems, psychiatric labels—which are stigmatized and inhibit help-seeking (8)—can be deemphasized. This is likely to result in a reduction and/or removal of stigma—which can be understood as de-stigmatization—toward people with mental health problems and an improvement in treatment seeking behavior for such problems. Moreover, it could be less emotionally encumbering to accept diagnosis and treatment plans which highlight two or three central nodes (most influential symptoms) as opposed to those which include stigmatized psychiatric labels.

Secondly, various studies show that common mood disorders such as MDD are expressed differently within different contexts (7, 15, 17). This is unsurprising because culture informs the experience of mental health disorders, and Western-derived taxonomies often miss culturally-salient features of distress (14, 16, 28). Accordingly, it can be quite beneficial to utilize the network approach to identify the dominant networks and most influential nodes which are specific to local cultural contexts.

Thirdly, by considering mental health disorders as interrelationships between specific symptoms rather than latent diseases, we can create new help-seeking avenues for mental health care across Africa. Currently, one hindrance to help-seeking is the length and cost of traditional therapies that often require delivery by expert mental health caregivers in regions with a paucity of such experts. Researchers have attempted to address this hindrance through successful trials of low-cost interventions that are delivered by lay-providers without the need for expert delivery (29, 30). Now, with the use of the network approach, researchers can identify the most influential mental health challenges amongst their target demographic and develop low-cost interventions targeting these specific challenges.

Finally, Western researchers still continue to dominate mental health research in low-and middle-income countries including those in African (31). We believe that multi-cultural collaboration where African researchers are empowered and spearhead research—dedicated to mental health—is important if we are to decolonize mental health. As such, the relatively novel network approach to psychopathology could encourage local researchers to spearhead research efforts and knowledge production and to pioneer treatment design. The increased involvement of local researchers could in turn promote community involvement in various parts of the research and treatment design processes, in addition to promoting help seeking.

In conclusion, it is important to emphasize that the network approach to psychopathology is intentionally flexible (24). As such, it is meant to be used as an “organizing framework” with which to understand existing data (24). Therefore, introducing this approach into African communities could help design research and treatment that is more contextualized, empowering, and effective through reducing stigma, encouraging local researchers, and boosting community involvement.

## 3. Discussion

Mental health problems present a significant global health priority around the world but specifically in low-resource regions like African countries. The consequences of these problems are devastating to individuals and communities. As such, there is an urgent need to transform mental health care for the better, for all.

Unfortunately, in Africa, efforts to transform mental health care are handicapped by the violent and colonial history of “formal” mental healthcare across the continent. Here, we show that because of this history, many individuals who struggle with mental health problems cannot access the help that they need and deserve. As such, efforts to make mental health care better can benefit from a decolonizing approach. Here, we present the network approach to psychopathology as a possible framework to decolonize mental health in Africa. Through the network approach, mental health disorders are conceptualized as complex interrelationships between network of symptoms as opposed to distinct disorders with western-derived psychiatric labels. This approach allows us to decolonize psychology because it alleviates stigma, allows for context-based understanding of mental health, opens new avenues for help seeking, and encourages local researchers to pioneer context-based knowledge production and treatments.

## Data availability statement

The original contributions presented in the study are included in the article, further inquiries can be directed to the corresponding author.

## Author contributions

RA and TO: perspective design, implementation, original draft manuscript, and manuscript revision. CW: perspective design and draft manuscript edits. All authors contributed to the article and approved the submitted version.
